# Thermo-Mechanical Analysis of Mass Concrete Foundation Slabs at Early Age—Essential Aspects and Experiences from the FE Modelling

**DOI:** 10.3390/ma15051815

**Published:** 2022-02-28

**Authors:** Aneta Smolana, Barbara Klemczak, Miguel Azenha, Dirk Schlicke

**Affiliations:** 1 Faculty of Civil Engineering, Silesian University of Technology, Akademicka 5, 44-100 Gliwice, Poland; barbara.klemczak@polsl.pl; 2 ISISE, Campus de Azurem, University of Minho, 4800-058 Guimarães, Portugal; miguel.azenha@civil.uminho.pt; 3 Institute of Structural Concrete, Graz University of Technology, Lessingstraße 25/1, A-8010 Graz, Austria; dirk.schlicke@tugraz.at

**Keywords:** early age concrete, foundation slabs, hydration temperature, thermal stress, finite element analysis (FEA)

## Abstract

In this paper, the focus is placed on essential aspects of finite element modelling of thermo-mechanical behaviour of massive foundation slabs at early ages. Basic decision-making issues are discussed in this work: the potential need to explicitly consider the casting process in the modelling, the necessary size of the underlying soil to be modelled and the size of the FE mesh, and the need of considering daily changes of the environmental temperature and the temperature distribution over the depth of the soil. Next, the contribution of shrinkage to early age stresses, the role of the reinforcement, and the type of mechanical model are investigated. Comparative analyses aiming to investigate the most important aspects of the FE model and some possible simplifications with negligible effect on the results are made on the example of a massive foundation slab. Finally, the results are summarized with recommendations for creating the FE models of massive slabs at early ages.

## 1. Introduction

Modelling of mass concrete at early ages using finite element analysis (FEA) has received a lot of attention among researchers. Some of them present the influence of the particular factors on the behaviour of the structure [1,2,3,4,5,6,7,8]; some are focused on the presentation of the original theoretical models, their implementation, and validation [9,10,11,12,13,14,15,16,17,18,19,20]. For the purpose of performing the FE analysis of concrete structures at early ages, usually, user-friendly commercial tools are available, for example, DIANA software, as in [21], where the thermo-hygro finite element framework for predicting the service life performance of reinforced concrete structures was presented, or MIDAS Civil software, which enabled performing the FE analysis of the temperature field in the arch bridge including the parametric analysis of the influence of the ambient temperature, cement type, and convection coefficient [22]. Moreover, some researchers used original programs and procedures dedicated to solving thermo-mechanical problems in early age mass concrete. Such an original finite element procedure for the chemo-thermal model based on the chemical affinity concept was applied in [23] to examine the temperature distribution of the dam during its construction. The original software packages TEMWIL (hygro-thermal analysis) and MAFEM (mechanical analysis) were used in investigations of the early behaviour of reinforced concrete tank walls [24] and slabs [5].

At this point, it should be mentioned that, despite the complexity of the theoretical model, all FE simulation models are only an approximation of the real structures and processes. There are two basic risks that the model produced is not an adequate approximation to the real problem. The source of the first one can be in the imperfect and too simplified theoretical description of the modelled phenomenon, including mainly the behaviour of the early age concrete and its hardening process. The second one can be directly related to the prepared finite element model and the accuracy of all actual material and technological factors. In recent years, due to computer capabilities, the tendency was to increase the number of introduced variables and the size of the model. Simultaneously, it increased the time required to produce the calculation model, even when full data were known. Therefore, efficient modelling of many phenomena using finite element analysis requires some inevitable simplification. Customarily, this simplification process is usually based on the modeller’s qualification to create a computation model with proper balanced complexity and accuracy.

The above-mentioned issues are particularly related to the modelling of concrete curing in massive foundation slabs. In this case, the challenging complex phenomena require FE modelling, like heat and mass flow due to the evolved cement hydration heat under conditions of variable temperature and humidity of the environment. Then, the stress analysis requires considering the evolution of concrete mechanical properties, which undergo phase changes during the hydration process, and finally, becomes a solid material. Therefore, the material model and the process itself are relatively complicated. Additionally, there are many other detailed data related to the specificity of the casting process as casting technology, environmental conditions, and the subsoil interaction. A general review of the early age properties of concrete, which is important for the evaluation of the cracking risk in mass concrete, has been recently presented in [25].

The performed literature review indicates that only a few studies are devoted to decision-making details in the adjustment of FE models for early age concrete structures. In this regard, a summarizing study of different thermo-mechanical models with the definition of the discrepancies between experimental results and numerical simulations is provided in [26]. A basic overview of the main approaches for modelling the behaviour of concrete at early ages and beyond is also provided in RILEM state-of-the-art report [27]. The investigation on the importance of some factors and numerical aspects in FE models has been recently presented in [28] for arch dams. Nevertheless, the FE modelling strategy is usually not thoroughly discussed and relies rather on the judgment of researchers performing these calculations, while some aspects involved in the FE analysis can be crucial.

The main aim of the following paper is to facilitate the process of modelling by the provision of the recommendations and guidelines for the FEA of massive foundation slabs at early ages. The focus of attention is paid to the essential aspects involved in thermo-mechanical FE modelling, such as the continuous process of casting of the slab, the size of interacting soil in the model, and the size of the finite element mesh, the need of considering the daily changes of the environmental temperature, and the real temperature distribution over the depth of the soil. Furthermore, the contribution of shrinkage to early age stresses and the role of the type of mechanical model are investigated as they may also affect the results of the FE model. The mentioned issues are usually assumed in the numerical model without a careful analysis of their impact on the results of the calculations. Thus, this work aims to analyse the most critical aspects in the FE thermo-mechanical modelling of mass concrete and show their effect on the obtained results. The applied FE model has been previously validated through the example of a real massive foundation slab. Next, this slab was used for the extended comparative analyses of the previously mentioned specific issues and their impact on the results of thermal-mechanical fields.

This paper is divided into six sections. After this introduction, Section 2 presents a brief description of the reference massive foundation slab used in the FE analysis. The applied computational model is described in Section 3. Next, in Section 4, the scope of the considered aspects in the FE modelling is described with the necessary data. The results obtained from the FE comparative study are presented and discussed in Section 5. Finally, conclusions and recommendations, which might be useful in the FE modelling of the mass foundation slabs, are provided in Section 6.

## 2. Case Study Description

The reference case for the FE analysis is the first construction stage of the massive foundation slab of the sluice Sülfeld-Süd in Germany [29,30]. The considered slab has a thickness of 2 m and dimensions of 26.5 × 41.5 m in a plan view. These dimensions, especially the thickness of the slab, are the reason for its consideration as a massive structure [31,32] with a significant rise in the concrete temperature caused by the cement hydration. In such structures, close-to-adiabatic conditions are observed in the core while the external surfaces are colder due to the heat transfer to the environment. Consequently, the interior of the mass concrete slab initially expands more than the external layers, and non-uniform thermal strains arise. The resulting stresses might gain significant values matching the actual tensile strength of the concrete and consequently cause cracking. Hence, this dictates the need for a careful study of this structure at the stage of concrete hardening, covering both the development of hydration temperature and induced stresses.

The concrete mix composition used in the slab was as follows [29,30]: cement CEM III/A 32.5 N (240 kg/m^3^), water (150 kg/m^3^), gravel aggregate: 0/2 mm (703 kg/m^3^), 2/8 mm (222 kg/m^3^), 8/16 mm (462 kg/m^3^), 16/32 mm (462 kg/m^3^), fly ash (110 kg/m^3^), BV 15 (Melius) 1.5% (3.6 kg/m^3^). The 28-day compressive strength of the applied concrete was 34.2 MPa, the tensile strength was 1.83 MPa and the modulus of elasticity was 34.4 GPa. In the FE analyses considering the effect of the reinforcement, at all external surfaces steel bars ϕ 25 mm at spacing of 15 cm and a concrete cover of 6 cm were assumed. The Young’s modulus of the reinforcement was assumed as equal to 210 GPa and the characteristic yield strength was 500 MPa.

**Figure 1:**
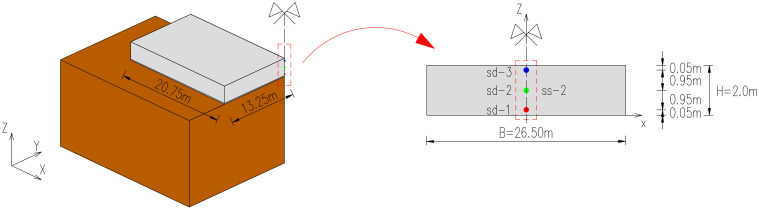
The locations of sensors in the slab: sd—vibrating wires for temperature, ss—stressmeter [29,30,33,34].

**Figure 2:**
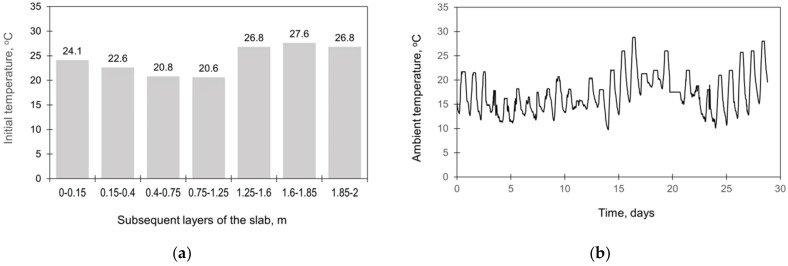
The registered initial temperature of the slab layers (**a**) and the ambient temperature (**b**) [29,30].

**Figure 3:**
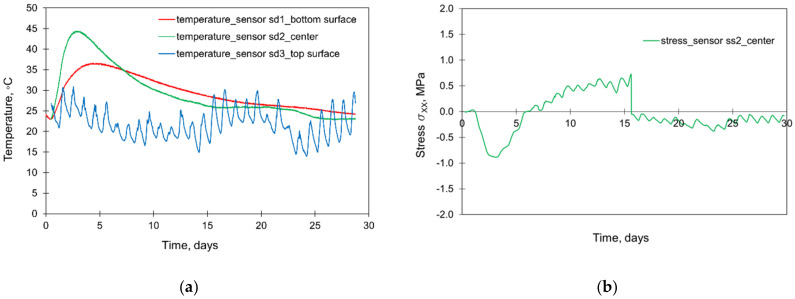
The results from the thermal (**a**) and stress (**b**) sensors [29,30].

The slab was cast continuously for 12.5 h in August 2005. The lateral surfaces were protected with the formwork for the first 7 days of concrete curing. During the construction process, the monitoring system covering the measurements of temperature, deformations, and stresses in the slab was applied [29,30]. The locations of the sensors are presented in Figure 1. The initial temperature of successively appearing concrete layers and ambient temperature were registered (Figure 2). The results from the thermal and stress sensors are depicted in Figure 3.

## 3. Computational Model

Difficulties with the modelling of the thermo-mechanical effects in mass foundation slabs are related to many factors influencing the magnitude and development of early age volume changes caused by cement hydration. Additionally, these changes occur with a simultaneous variation of mechanical properties. Therefore, the FE model requires the determination of nonlinear transient thermal and stress fields considering the cement hydration, developing concrete properties, and environmental impacts.

For thermal analysis, assuming concrete as a continuum isotropic material with the internal heat source, the differential equation for energy balance is used in the following form [35]:

(1) $$\rho c_{p}\frac{\partial T}{\partial t}=\lambda \left(\frac{\partial^{2}T}{dx^{2}}+\frac{\partial^{2}T}{dy^{2}}+\frac{\partial^{2}T}{dz^{2}}\right)+\dot{q}$$

where *T*—temperature (°C), ρ—density of concrete (kgm^−3^), $c_{p}$—specific heat (Jkg^−1^°C^−1^), *t*—time (s), λ—thermal conductivity (Wm^−1^°C^−1^), *x*,*y*,*z*—coordinates (m), $\dot{q}$—rate of internal hydration heat generation (Wm^−3^), calculated according to the formula:

(2) $$\dot{q}=f\left(\alpha_{T}\right)A_{T}e^{\frac{-E_{a}}{RT}}$$

where $f\left(\alpha_{T}\right)$—normalized heat generation rate, $A_{T}$—rate constant, *R*—universal gas constant (8.314 Jmol^−1^°C^−1^), $E_{a}$—apparent activation energy (Jmol^−1^).

The heat transfer between the concrete and the environment is considered by a simplified, lumped coefficient α, representing both convection and radiation [27,36]. The Neumann-type boundary condition is used for the determination of heat transfer from the outer surfaces:

(3) $$q_{eq}=\alpha \;\left(T_{surf}-T_{env}\right)$$

where $q_{eq}$—the heat flux from the boundary (Wm^−2^), α—the lumped convection-radiation heat transfer coefficient (Wm^−2^°C^−1^), $T_{surf}$—the temperature of the boundary surface of the element (°C), $T_{env}$—the environmental temperature (°C).

The convection-radiation coefficient, α, is taken as a sum of the natural convection, $\alpha_{n}$, the forced convection due to wind action, $\alpha_{f}$, and the radiation, $\alpha_{r}$:

(4) $$\alpha =\alpha_{n}+\alpha_{f}+\alpha_{r}$$

The natural convection was assumed as equal to 6.0 Wm^−2^°C^−1^ [36], and the forced convection due to wind action was taken as a function of wind speed v [36]:

(5) $$\alpha_{f}=3.7\;v$$

Generally, the radiation emitted by a given body is described by the Stefan–Boltzmann law, according to which a material emits radiation at a rate proportional to the fourth power of its absolute temperature *T*. In the presented study, a constant value of $\alpha_{r}$ = 5.2 Wm^−2^°C^−1^ is taken, as proposed in [37].

In the mechanical part, a viscoelastic material model with temperature-dependent Young’s modulus has been applied in the analysis presented in Section 5.1, Section 5.2, Section 5.3, Section 5.4, Section 5.5, Section 5.6. Basic creep of concrete is considered with the application of the Double Power Law [38]:

(6) $$J\left(t,t^{\prime }\right)=\frac{1}{E\left(t^{\prime }\right)}+\frac{\phi_{1}}{E\left(t^{\prime }\right)}{\left(t^{\prime }\right)}^{-m}{\left(t-t^{\prime }\right)}^{n}$$

where $J\left(t,t^{\prime }\right)$—compliance function (GPa^−1^) at time *t* (days) for a load applied at instant *t*′ (days), $E\left(t^{\prime }\right)$—asymptotic Young’s modulus (GPa) of concrete at each loading age *t*′, $\phi_{1}$, *m*, *n*—material parameters. 

The combined effect of temperature and time on the evolving Young’s modulus is considered using the equivalent age approach. The equivalent age represents the concrete age at the reference curing temperature that would result in the same properties as would result from curing at other temperatures. Thus, the influence of elevated curing temperatures on the development of Young’s modulus, *E*, is considered by introducing equivalent time $t_{eq}$ instead of time *t*′ [39,40]:

(7) $$E\left(t^{\prime }\right)=E\left(t_{eq}\right)=\alpha_{1}e^{-{\left(\frac{\tau_{1}}{t_{eq}}\right)}^{\beta_{1}}}+\alpha_{2}e^{-{\left(\frac{\tau_{2}}{t_{eq}}\right)}^{\beta_{2}}}$$

where $\alpha_{1}$, $\alpha_{2}$ in GPa and $\tau_{1}$, $\tau_{2}$ in days, $\beta_{1}$, $\beta_{2}$ (unitless)—parameters determined based on measurements of Young’s modulus at curing temperature equal to 20 °C.

The equivalent age, $t_{eq}$, considering the influence of elevated temperature of concrete curing, is calculated as follows [41]:

(8) $$t_{eq}=\overset{t}{\underset{0}{\int }}e^{-\frac{E_{a}}{R}\left(\frac{1}{T\left(\tau \right)}-\frac{1}{T_{ref}}\right)}d\tau$$

where *T*(*τ*) stands for the actual concrete temperature (°C), $T_{ref}$ is the reference temperature equal to 20 °C, *R* stands for universal gas constant (8.314 Jmol^−1^°C^−1^), $E_{a}$ is the activation energy (Jmol^−1^).

Additionally, another material model of young concrete has been also used (Section 4.7 and Section 5.7), enabling the analysis of crack development in early age concrete members. In this case, cracks are simulated with a multi-directional fixed crack model [40]. For reinforcement, an elastic-perfectly plastic model is applied. More details connected with the applied crack-based model are given in [32,33,40].

Furthermore, the linear elastic isotropic model was applied for the subbase (lean concrete) and the soil. The comparative FE analysis was performed with the use of DIANA IE 10.2 software which is dedicated to a wide range of civil engineering problems. The analysis of concrete slabs at early age consists of two steps: the first was to simulate the temperature history due to the cement hydration and the second one was to simulate the development of induced stresses. The influence of the thermal fields on the mechanical fields is manifested by considering the impact of increased hardening temperature on the development of mechanical properties, i.e., the modulus of elasticity and tensile strength used in the failure criterion. Therefore, the applied model can be identified as one-way coupling.

## 4. The Scope and Data for FE Simulation

The thermo-mechanical model addressed in Section 3 was applied to study a massive foundation slab described in Section 2. In Section 4.1, the necessary data used for the validation of the computational model are presented. For comparative purposes, further analyses of essential aspects involved in the thermo-mechanical FE modelling of foundation slabs at early ages were performed. These subsequent analyses are based on the same slab, which is described in Section 2 and Section 4.1. The explanations provided in Section 4.2, Section 4.3, Section 4.4, Section 4.5, Section 4.6 and Section 4.7 are directed to the considered modelling options in the FE analysis. Then, their impact on the thermal-mechanical fields is examined with the identification of the most critical aspects for the FE simulation of the massive foundation slab.

### 4.1. Basic Data for the FE Validation of the Case Study

**Figure 4:**
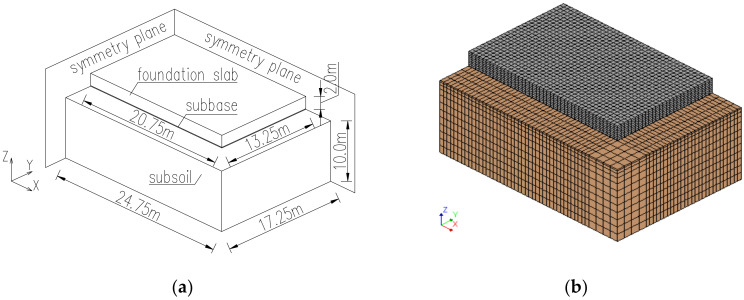
The geometry of the ¼ of the analysed structure: (**a**) dimensions, (**b**) the FE model [32,33,34,42].

The geometry and FE model of the analysed slab is presented in Figure 4. Aiming for the diminution of the number of finite elements, the size of the FE model was reduced to ¼ of the structure by using symmetry conditions. The twenty-node solid brick finite elements have been applied in the model. It is based on quadratic interpolation and Gauss integration. In the planes of symmetry, the fixed supports are applied in the model. At the top surface of the soil, no supports are applied. At the lateral surfaces of the model, the horizontal movement is restricted, while at the bottom surface the vertical movement is blocked.

Material properties assumed in the study are based on the data provided in the report from measurements [30] and dedicated literature [29,43,44]. The proper values for slab concrete, subbase, soil, and reinforcement used in the computations are listed in Table 1.

The particular values in Equation (2) as the rate constant $A_{T}$ = 1.75∙10^9^ Jm^−3^s^−1^ and the apparent activation energy $E_{a}$ = 38,500 Jmol^−1^ were derived from adiabatic data [29,33].

The following thermal boundaries were applied in the FE model: for all unprotected surfaces (slab and subsoil), the convection-radiation coefficient is taken as 30 Wm^−2^°C^−1^ based on Equation (4) with the assumed wind speed 5 ms^−1^. For lateral surfaces of the slab, covered with a plywood formwork 22 mm thick at first 7 days after casting (with thermal conductivity of 0.14 Wm^−1^°C^−1^), the electrical analogy detailed in [24,37] was used to calculate the convection-radiation coefficient, which takes the value of 5.2 Wm^−2^°C^−1^. For the lateral surfaces of the subsoil and the surfaces in the planes of the symmetry, the adiabatic conditions were applied.

In the empirical Equation (6), the coefficients were based on [29,33] and adapted for the sluice’s concrete by adjustment of the particular curves corresponding to the data provided in [5,29,30]. Considering $E\left(t^{\prime }\right)$ in GPa and the compliance function $J\left(t,t^{\prime }\right)$ in GPa^−1^, the following values are obtained: $\phi_{1}$ = 0.012, *n* = 0.263, *m* = 0.016. Similarly, in the empirical Equation (7) parameters determined from measurements are as follows: $\alpha_{1}$ = 15 GPa, $\tau_{1}$ = 2 days, $\beta_{1}$ = 1.5, $\alpha_{2}$ = 20 GPa, $\tau_{2}$ = 4 days, $\beta_{2}$ = 1.5. Hence, for the reference concrete, temperature equals 20°C, and the evolution of Young’s modulus is in GPa; the result from Equation (7) fits the experimentally registered value of the 28-day modulus of elasticity equal to 34.4 GPa (Table 1). In Equation (8) the apparent activation energy $E_{a}$ = 38,500 Jmol^−1^ was applied, similarly as in Equation (2).

In the validation of the model (later denoted as ‘validation’), the thickness of the slab was divided into 7 layers of different thicknesses to reproduce the process of the actual continuous casting.

**Figure 5:**
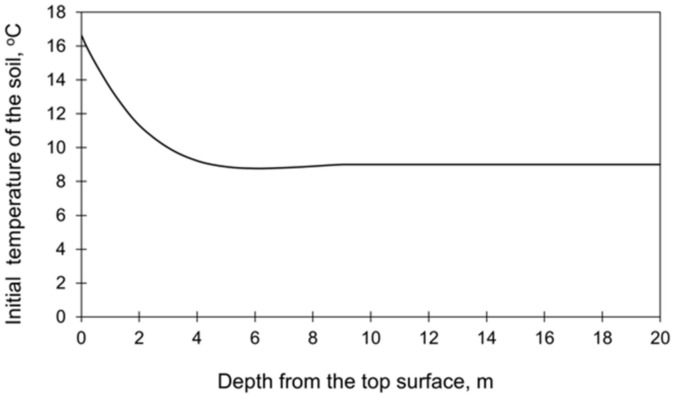
The initial temperature of the subsoil [33,45].

Table 2 presents the start times, stepping strategy, and thickness of each layer. The initial temperature of each layer of the slab as well as the ambient temperature were taken based on the measurements (Figure 2). The initial temperature profile in the subsoil, presented in (Figure 5, was determined based on the preliminary FE analysis [33,45]. The total time of the performed analysis was 28 days.

### 4.2. The Slab Casting Process 

In this study, the need to model a continuous process of casting of the slab in the FE simulation was investigated. A detailed scheme of the continuous casting of the slab applied in the validation of the FE model (Table 2) has been compared with the model omitting this process and assuming placing of the concrete in the slab at ‘one time’. Such an approach, undoubtedly simplified, significantly shortens the time of data preparation for the model, and it seems interesting how it affects the results of calculations. The same material data and the FE mesh as for the validation (Section 4.1) were applied in this study. The initial temperature of concrete was assumed to 24 °C, as a mean value from the registered temperature of each subsequent layer (Figure 2). Additionally, a slab with the same plan view dimensions but with a thickness of 4 m was also examined.

### 4.3. Computational Domain

The foundation slabs are usually large in their dimensions and a significant number of finite elements can be generated. Furthermore, it is necessary to consider the subsoil interaction in the FE simulation. The result is a large FE model with long computation times. Hence, two issues are investigated in this part of the comparative FE analysis:

1. The size of the finite element mesh applied in the slab structure and its impact on the temperature and stress development,
2. The adequate size of interacting soil in the FE model, not affecting the thermal and stress development.

Considering the first issue, it is known that the accuracy of the finite element analysis as an approximation method is determined by the mesh size. A dense mesh significantly increases the time of analysis; thus, the optimal selection of the FE size is crucial during the pre-processing of the model. The effect of the mesh density on the numerical results was the subject of many studies [46,47]. Moreover, guidelines might be found in the manuals of particular software [48,49]. Nevertheless, the adoption of the finite element mesh usually stands on the researcher or practitioner performing the FE simulations. This part of the pre-processing of the model is especially important in foundation slabs, of usually large dimensions and, consequently, a great number of finite elements. 

**Figure 6:**
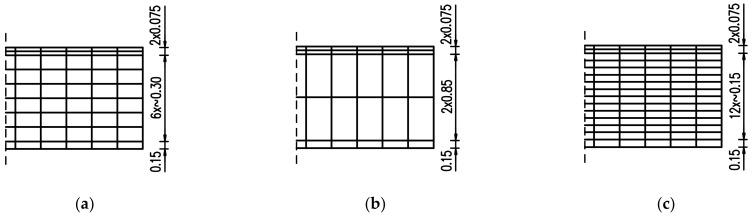
The height of finite elements at the thickness of 2 m slab: (**a**) based on Figure 4b—validation case, (**b**) coarse mesh, (**c**) dense mesh.

**Figure 7:**
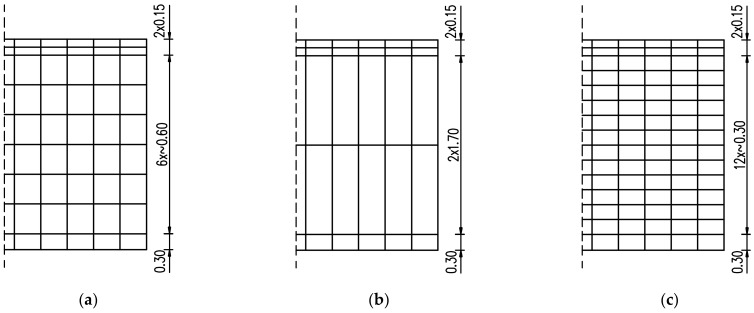
The height of finite elements at the thickness of 4 m slab: (**a**) basic case, (**b**) coarse mesh, (**c**) dense mesh.

Thus, the optimal FE mesh and its influence on the development of hardening temperature and stress in the slab were examined. In this regard, both reduction and densification of the initial mesh (Figure 4b) at the thickness of the slab have been applied as presented in Figure 6. The slab with a thickness of 4 m is also considered (Figure 7). The finite element mesh at the top surface in the examined slabs was the same due to the effects of boundary conditions. The same material data as for the validation (Section 4.1) were applied, except the casting process which was assumed at ‘one time’, as described in Section 4.2.

In the second part of the comparative analysis dedicated to the computational domain, the different sizes of the cooperating soil were compared. Obviously, the smallest size of the subsoil is preferred due to the number of finite elements in the model. Therefore, it has been examined how the vertical and horizontal sizes of the subsoil interfere with the obtained thermal and stress results. The vertical dimension of the subsoil was evaluated in the preliminary FE study [33,45]. The results showed that a depth of 10 m is sufficient and does not affect the temperature and stress distributions in the slab. It is also the depth below which the initial ground temperature is stable (Figure 5). 

**Figure 8:**
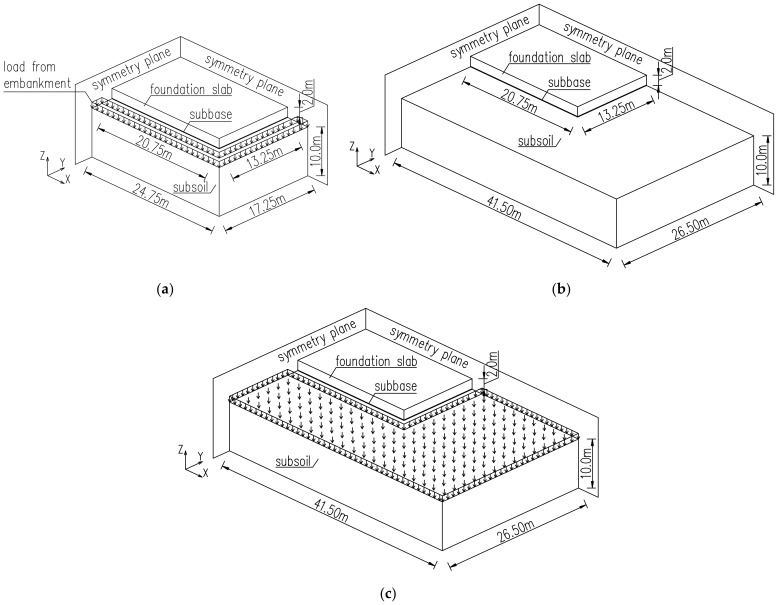
Study on the subsoil modelling: (**a**) case ‘load’, (**b**) case ‘long’, (**c**) case ‘long_load’.

It is also reasonable to reduce the horizontal dimensions of the interacting soil due to the limitation of the number of finite elements and consequently the time of analysis. Thus, the key issue pertains to the subsoil dimensions which should be included in the finite element model. In this regard, the validation case (Section 4.1) with the soil dimensions in the plan view of 4 m longer in the directions X and Y than the above-laying slab (Figure 4a) has been compared with three additional FE models. In the first case, the top surface of the soil was loaded with the weight representing the embankment, which is usually omitted in FE simulations although usually the foundation slabs are made in an excavation (Figure 8a). The second case considered the dimensions of the soil in the plan view assumed as the double-length/width of the slab (Figure 8b), while the last case represented the longer dimensions in the plan view with the load representing the embankment (Figure 8c). The remaining data in this study were taken as for the validation case (Section 4.1).

### 4.4. Effect of the Environmental Temperature

In the validation case (Section 4.1) the actual changes of the environmental temperature throughout the 28 days of concrete curing were applied in the FE model. Nevertheless, most of the FE simulations used a fixed value of the environmental temperature, which is a simplified but less troublesome approach. In this regard, introducing the real environmental temperature to the model, different for each time step in the analysis, can be time-consuming in some software, especially if it is required to adjust the set ambient temperature changes to the assumed calculation steps. Additionally, in the prediction analysis, only the forecasted data are available, and thus, it is hard to predict the real temperature conditions in the design phase. Therefore, the defined safe-side scenarios for the ambient temperature will be beneficial for the simulation of the slabs. 

The main objective of this study is to assess the differences in temperature and stress developments simulated using the model including real changes of the ambient temperature throughout the analysed period with the simplified approach assuming constant ambient temperature. The validation case (Section 4.1) with the actual ambient temperature registered during operation (Figure 2b) was compared to the different values of the fixed temperature:

- the average ambient temperature throughout the analysed 28 days, equal to $T_{env}$= 18 °C;
- the minimum ambient temperature occurring in this time, equal to $T_{env}$= 11 °C;
- the maximum ambient temperature occurring in this time, equal to $T_{env}$= 28.8 °C.

The remaining data in this study was taken as for the validation case (Section 4.1). The FE analysis was extended also to a slab of 4 m thick, using the same material and technological data.

### 4.5. Properties of the Soil

Two additional soil-related options were investigated in the FE comparative study. The first concerned the significance of the precise reproducing of the initial temperature profile over the depth of the soil in the FEA while the second was related to the importance of the actual soil stiffness.

**Figure 9:**
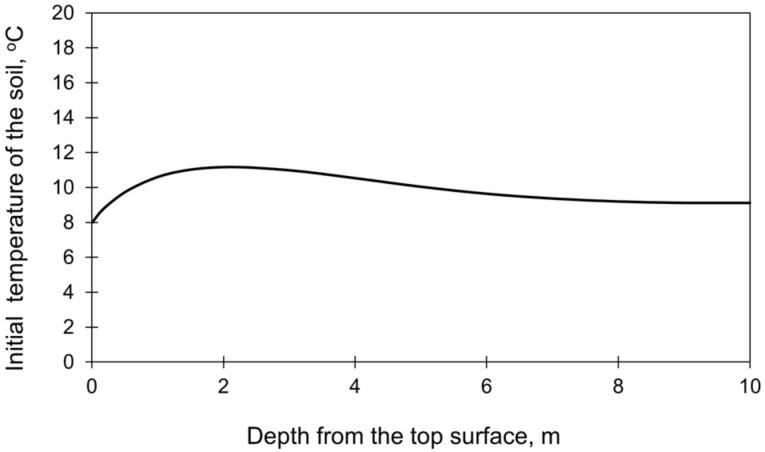
Initial temperature distribution at the soil depth in the autumn/winter seasons [33,45].

Considering the first issue, the analysis of the mass foundation slab requires assigning the initial temperature not only to the concrete slab but also to the underlying ground. The temperature of the soil depends on the environmental temperature, and it is not constant over its depth. This fact can be considered in two ways. In the first approach, an additional calculation step in which the FEA is performed only for the soil using the actual ambient temperature. The second approach is based on the analytical calculations of the temperature distribution in the soil [45,50,51]. Then, the obtained temperature distribution over the soil depth is set as the initial soil temperature in the FE model of the whole structure. In both cases, feeding such input to the model results in both additional time and preliminary calculations. In this context, the assumption of constant soil temperature in the FE model would be much more convenient and less time-consuming. Thus, the models with the diversified and constant initial temperature over the soil depth have been compared to assess the importance of the assumed initial soil temperature. Two casting seasons were analysed in this regard:

1. the validation case (Section 4.1) related to the summer season was compared with the FE model with the assumed constant temperature of the soil equal to 16 °C;
2. the autumn/winter casting conditions with varying temperature distribution at the soil depth (Figure 9) were compared with the constant temperature equal to 8 °C.

In the validation case, the soil with low stiffness was applied (Table 1). To verify the importance of the proper soil stiffness in FEA, the soil with higher stiffness with E-modulus equal to 100 MPa was also examined.

All data in this investigation were taken as for the validation case (Section 4.1), except the environmental temperature in the autumn/winter seasons assumed as a fixed value of 8°C because of the absence of precise data.

### 4.6. Effect of Shrinkage

In the validation case, shrinkage deformations have been disregarded as in massive foundation slabs, they are generally considered to have a negligible effect on stresses. To assess the validity of this frequently used simplification, the FE analysis of the slab including drying shrinkage deformations was additionally performed. The shrinkage strains were computed using the moisture model [37,40], based on the averaged pore relative humidity $H_{c}$ as a driving potential. Next, the transformation of the computed moisture field $H_{c}$ into a field of unrestrained potential shrinkage is described by the following equation [37]:

(9) $$\varepsilon_{sh,pot}=\varepsilon_{sh,\infty }\left(0.97-1.895{\left(H_{c}-0.2\right)}^{3}\right)$$

where $\varepsilon_{sh,\infty }$ is the ultimate concrete shrinkage equal to 364 µε, estimated for 60% RH. 

**Figure 10:**
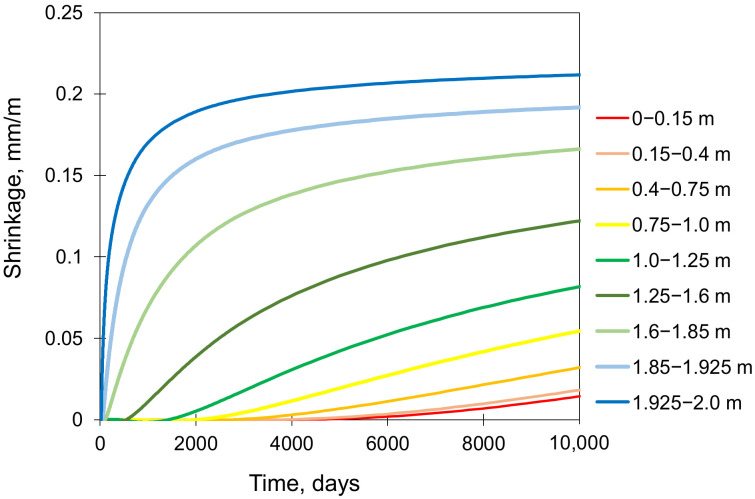
Potential drying shrinkage in the successive layers along with the slab thickness.

Afterward, the unrestrained potential shrinkage was subsequently implemented in the mechanical model as an imposed pre-strain to the particular layers of the slab. The time of the shrinkage analysis was extended to 365 days to examine this effect also in the later period. Figure 10 shows the potential free shrinkage evolutions along the slab thickness resulted from the computed pore relative humidity $H_{c}$, which have been applied as input to the mechanical model. The obtained curve evolutions clearly show the tendency of concrete to progressively shrink from the surface to the core.

### 4.7. Effect of the Material Model

The material model applied in FEA has a crucial impact on predictions of concrete behaviour. Generally, in most FE analyses of massive concrete structures, the viscoelastic material model without the crack simulation is used. Hence, the viscoelastic model used in the validation case was compared to the crack-based model mentioned in Section 3. Additionally, the influence of the surface reinforcement on the cracking behaviour of the slabs was examined. All above-mentioned issues were investigated for the data from the validation case (Section 4.1) with the casting process modelled as ‘one time’ (Section 4.2). The study was extended to a slab with a thickness of 4 m, using the same data.

## 5. Results and Discussion

**Figure 11:**
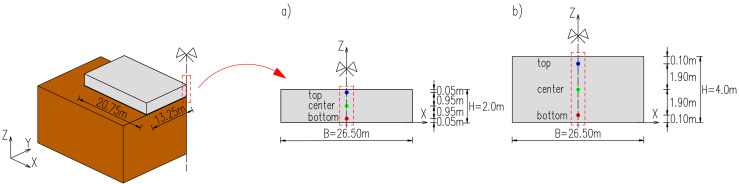
Locations of the reference points in the slabs of the thicknesses of (**a**) 2 m, (**b**) 4 m.

The results of the comparative analysis depicted in Section 4 have been presented for three selected points in the slab, located in the intersection of its vertical planes of symmetry. In the case of massive concrete structures, the main objective of the FE analyses is to assess the developing stresses and the risk of early-age cracking because it may reduce the later load-bearing capacity of the structure. The thermal fields are a load on the structure, and their determination is an indirect task here. Nevertheless, the temperature development is always carefully analysed because it can be relatively easy compared with the results of in situ measurements, which is the first stage of model validation at the load checking level. Hence, the crucial outputs of the FE thermo-mechanical modelling are both temperature and stress developments. Considering the obtained results, both hardening temperature and induced $\sigma_{xx}$ stress developments have been analysed for 28-days of concrete curing. In each case, the difference between different modelling options was presented, but in general, we did not try to rate the discrepancies at a percentage level. Where the discrepancies between the respective models were qualitatively different or quantitatively different by more than 10%, we assessed the impact as significant. Additionally, we focused our attention primarily on the maximum hardening temperature and maximum tensile stresses in the heating phase, which is the most important for the possible cracking. The typical sign convention for stresses induced in the slabs has been used in the presentation of the results. Hence, tension is positive, and compression is negative. The precise locations of reference points are given in Figure 11. 

The values of temperature have been read for relevant adjacent nodes. Next, assuming a linear change of its value, the proper temperatures were determined for three selected points shown in Figure 11. In the case of stresses, the stress values for the top elements have been taken. The heights of these elements are 7.5 cm (2 m slab) and 15 cm (4 m slab). The reference points are related to the 5 cm from the top surface (2 m slab) and 10 cm (4 m slab). Thus, they are included in the considered finite elements. Additionally, at the beginning, we checked the stress distributions in the exemplary cross-sections, and the values of the stress based on the linear interpolation of stresses between the adjacent finite elements for the points located at 5 cm and 0.5 × 7.5 cm = 3.75 cm (the centre of the finite element) did not differ by more than 0.05%. The same were obtained for the slab with a thickness of 4 m.

### 5.1. Validation of FE Model

**Figure 12:**
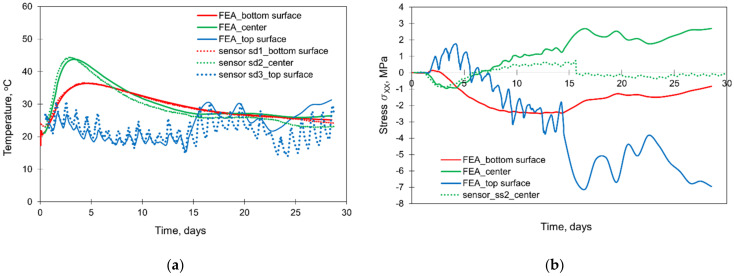
Validation of the FE model: (**a**) temperature development, (**b**) stress development [32,33,34,42].

The validation of the FE model of the structure has been already included in previous studies [32,33,34,44]. However, for the completeness of the considerations presented in this article, the compact results of the validation are depicted in Figure 12. Based on Figure 12a, quite good compliance was obtained in the temperature development generated by the hydration process. The difference in the maximum interior temperature obtained from the simulation differs from the measurement results by only 1°C. Considering the stress development (Figure 12b), good compliance between the measurement and FE model is visible by the 8th day of concrete curing. Later, the convergence becomes worse. In [32,34], it was explained by cracking occurring in an area close to the stressmeter’s location and affecting both the functionality of this sensor and the stress distribution in its surrounding. Nevertheless, the good coincidence in the first few days of concrete curing, as well as the correct stress inversion in the cooling phase, may be considered as a reliable FE simulation of all stresses.

### 5.2. Modelling the Casting Process in FEA

**Figure 13:**
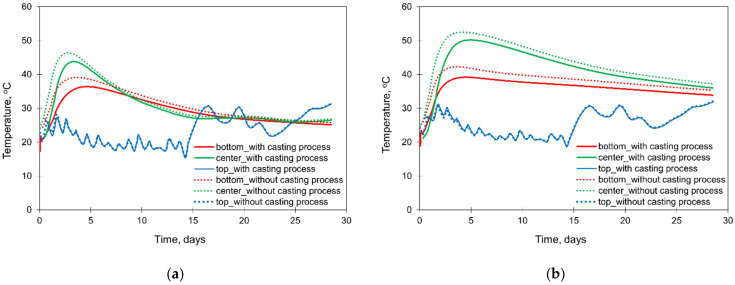
The influence of the casting modelling on the development of hardening temperature in the slabs with the thicknesses of (**a**) 2 m and (**b**) 4 m.

**Figure 14:**
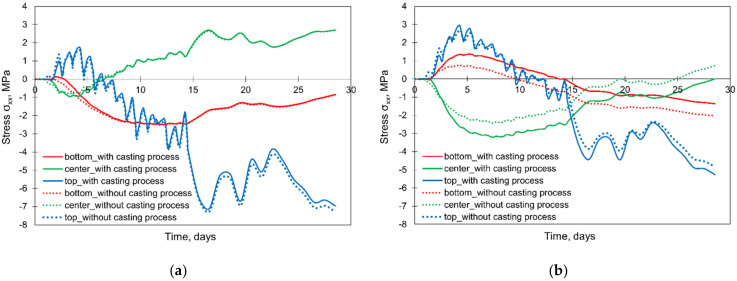
The influence of the casting modelling on the development of stress $\sigma_{\mathrm{xx}}$ in the slabs with thicknesses of (**a**) 2 m and (**b**) 4 m.

The results of the study are presented in Figure 13 (temperature development) and Figure 14 (thermal stress development). First of all, the method of modelling the casting process in FEA affects the hardening temperatures in the centre and at the bottom of the foundation slabs. For these two points, the calculated temperatures are higher in the simplified model without simulation of the casting process, which is perspicuous because then the successive cooling of subsequent layers of the slab is not modelled. The discussed differences are greater for the thicker slab (4 m). At the same time, the method of modelling the casting process does not affect the temperature development at the slab top surface (Figure 13a,b).

Considering the stress development (Figure 14a,b), noticeable differences were obtained for the centre and bottom surfaces of the thicker slab (4 m). The simplification of the casting process in the FE model causes the reduction of the initial peak of compressive stress by about 1 MPa in comparison to the case in which the accurate mapping of the slab casting was implemented (Figure 14b). The differences in the stress at the top surface of the slab are more complex. The model without the precise mapping of the casting process provides the underestimated results of the maximum tensile stress. In detail, in the case of the slab with the thickness of 4 m, it is 2.69 MPa (without the precise mapping of the casting process) and 2.96 MPa (when modelling the casting process). Simultaneously, in the earlier period about the 2nd day of concrete curing, higher values of the tensile stresses at the top surface are visible from the model not considering the casting process (Figure 14a,b).

At this point, it should be recalled that the main purpose of the FE analyses of early age slabs is to assess the risk of possible cracking. Such cracking risk in the foundation slabs refers to two scenarios, as described in [8,52], and whereby the higher risk arises in the heating phase when possible cracks may be induced at the top surface of the slab. From this point of view, higher values of the maximum stresses were obtained in the accurate mapping of the slab casting process. Thus, the precise reproduction of the casting process should be recommended, especially in thicker slabs, although it is more laborious in the preparation of the FE model.

### 5.3. Effect of the Various Computational Domains

**Figure 15:**
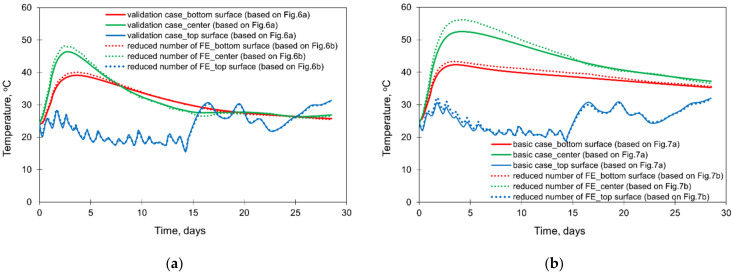
The effect of the different size of FE at the thickness of the slabs on the temperature development in the slab with thicknesses of (**a**) 2 m and (**b**) 4 m.

**Figure 16:**
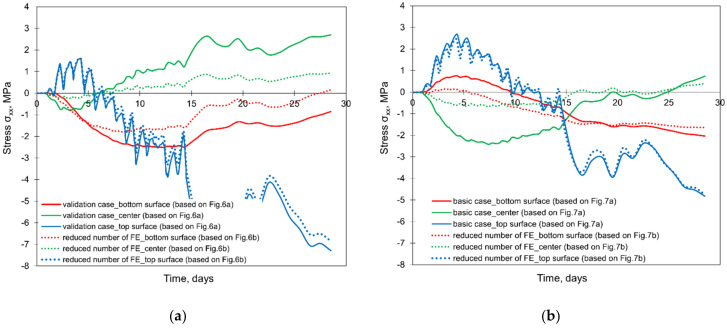
The effect of the different sizes of FE at the thickness of the slabs on the developments of stress $\sigma_{\mathrm{xx}}$ with thicknesses of (**a**) 2 m and (**b**) 4 m.

**Figure 17:**
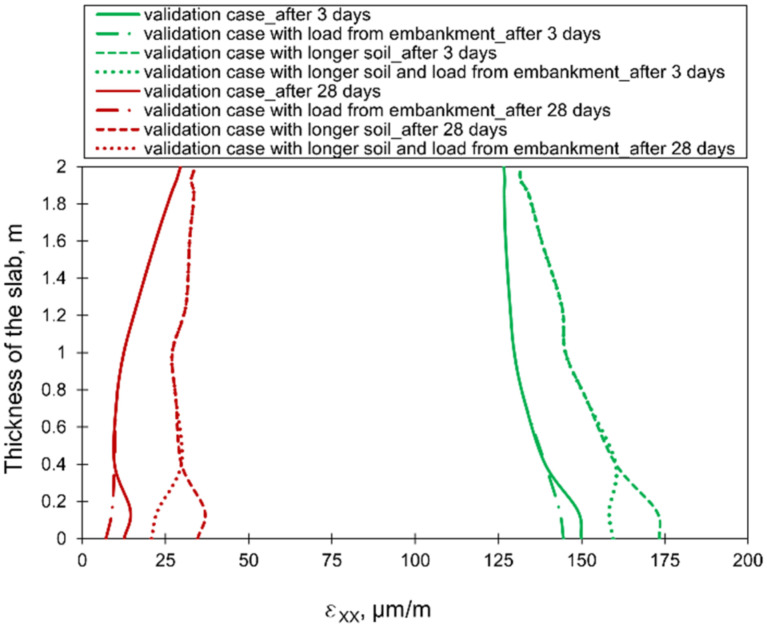
The effect of the different sizes of the cooperated soil on the distributions of strains $\varepsilon_{\mathrm{xx}}$ at the thickness of the slab.

The results of the comparative FE simulations are visible in Figure 15 and Figure 16 (the effect of the different sizes of FE at the thickness of the slab), as well as in Figure 17 (the effect of the different sizes of the cooperated soil).

Based on the presented results, the significant influence of the FE mesh size is observed only when an enormous reduction of the number of elements was applied (presented in Figure 6b and Figure 7b). The application of the smallest mesh size at the thickness of the slabs (presented in Figure 6c and Figure 7c) did not affect the temperature and stress development (Figure 15 and Figure 16). As the decrease of the mesh size does not reduce the accuracy of the model (has no meaningful influence on analysed temperature and stress values in the slab) but increases the number of the finite elements and the time of analysis, the following smallest required size of the mesh in the vertical direction is proposed:

(10) $$min\;h_{e}\left(z\right)=\left(0.15÷0.20\right)h$$

where: $min\;h_{e}\left(z\right)$—minimum mesh size in the vertical direction (thickness of the slab), in m, *h*—thickness of the slab, in m. Simultaneously, the proposed vertical minimum mesh size refers to the central part of the slab with the required net density at the top surface due to the boundary conditions. Concerning the stress analysis, it should also be emphasized that the presented results and conclusions relate to the analysis using the viscoelastic model for early age concrete.

Considering the different sizes of the cooperating soil, no significant influence of the soil horizontal dimension on the distribution and stress values was detected. This result was surprising; hence, a deeper analysis was carried out with the analysis of strain distributions. According to Figure 17, the different values of strains $\varepsilon_{xx}$ in the slab with the different sizes of the interacting soil are observed. The increase of the soil dimensions (case with longer soil presented in Figure 8b) caused the higher strains in comparison to the model with the smaller size of cooperating soil (validation case presented in Figure 8a). These differences concern mainly the bottom part of the slab, and gradually decrease towards the top surface (Figure 17). At the same time, the weight of the embankment affects only slightly the strain values at the bottom surface of the slab. An interesting issue is the difference in obtained strains, with the simultaneous lack of differences in stresses. It may be explained by the low Young’s modulus of concrete in the initial period of concrete hardening, which, despite the difference in strains, results in a negligible change in stresses. Later, the modulus of elasticity increases, but the strain values are relatively small (Figure 17).

Hence, to create an accurate finite element model for the simulation of the behaviour of the foundation slab, the horizontal size of the soil should be taken with particular care. Suggested action in this area is the preliminary calculations focused on the determination of the soil of the possible smallest size, which gives the same values of strains as for the longer interaction soil.

### 5.4. Effect of the Environmental Temperature

**Figure 18:**
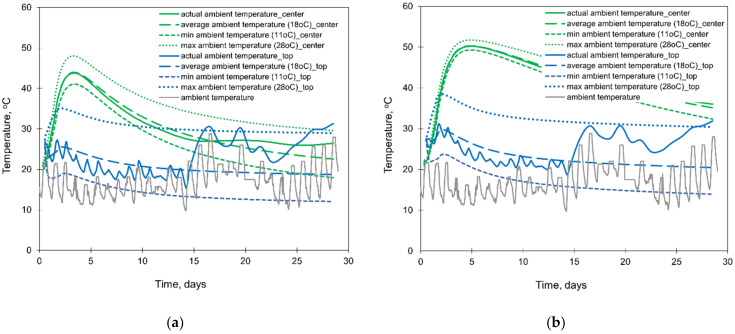
The effect of the ambient temperature on the temperature developments in the slabs with thicknesses of (**a**) 2 m and (**b**) 4 m.

**Figure 19:**
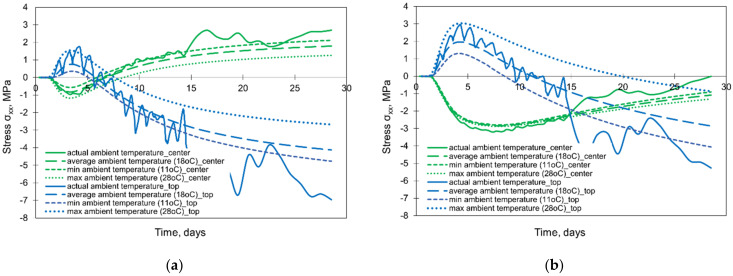
The effect of the different sizes of FE at the thickness of the slabs on the developments of stress $\sigma_{\mathrm{xx}}$ in the slabs with thicknesses of (**a**) 2 m and (**b**) 4 m.

The results of the numerical simulations are presented in Figure 18 and Figure 19. The depicted graphs indicate a significant role of the ambient temperature in the FE analysis of foundation slabs at early ages. The assumption of the constant ambient temperature significantly affects the surface cooling conditions of the slab, and as a result, the greater impact is noticeable in the temperature development at the top surface of the slab. Comparing the temperature in the centre of the slab, the thinner slab (2 m) is more sensitive to the ambient temperature (Figure 18). The influence of the ambient temperature on the temperature development at the bottom is negligible, and therefore, the graphs for this point were omitted.

Analysing the stress development, the substantial discrepancies between stresses are visible at the top surfaces (Figure 19). The maximum tensile stresses matching to the maximum values obtained from the FEA including real changes of the ambient temperature are visible for the model assuming the maximum ambient temperature (28.8 °C). This is an important observation for the reliable FE predicting cracking risk in the slab. However, in this case, the inversion of the stress occurs significantly later than in the case with the actual ambient temperature. Although the calculations with the assumed average outside temperature (18 °C) gave the temperature distribution similar to the precise model with actual ambient temperature, the stresses obtained under this assumption fit into the lower values of the precise model with the actual ambient temperature. Considering the centre of the 2 m thick slab, stresses differ slightly while in the 4 m thick slab they are practically the same. Hence, regarding the cracking risk, depending mainly on the maximum tensile stresses in the heating phase, it can be concluded that forecast daily temperature changes (day/night) should be considered in the FE analysis because of the significant impact on the results.

### 5.5. Effect of the Soil Properties

**Figure 20:**
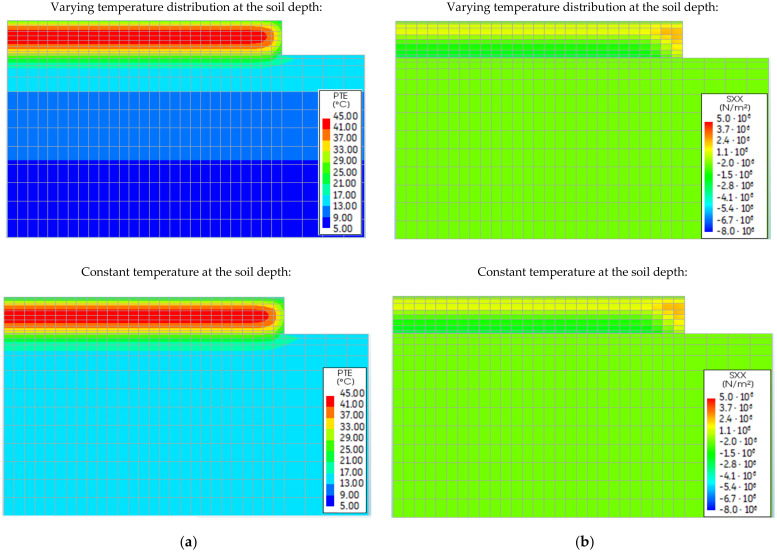
The effect of the initial soil temperature on the distributions of (**a**) temperature and (**b**) stress $\sigma_{\mathrm{xx}}$.

Figure 20 shows the contours of the temperature (Figure 20a) and stress (Figure 20b) in the heating phase, obtained for the summer season of the slab casting. Based on the presented figures, the assignment of the constant initial temperature of the soil in the FE model does not affect the distributions of temperature and stresses in the slabs. The same lack of significance was obtained for the case of the slab casting in winter conditions. Thus, the performed study shows that the assumption of a constant initial temperature of the subsoil does not reduce the accuracy of the FE analysis of the slab behaviour.

**Figure 21:**
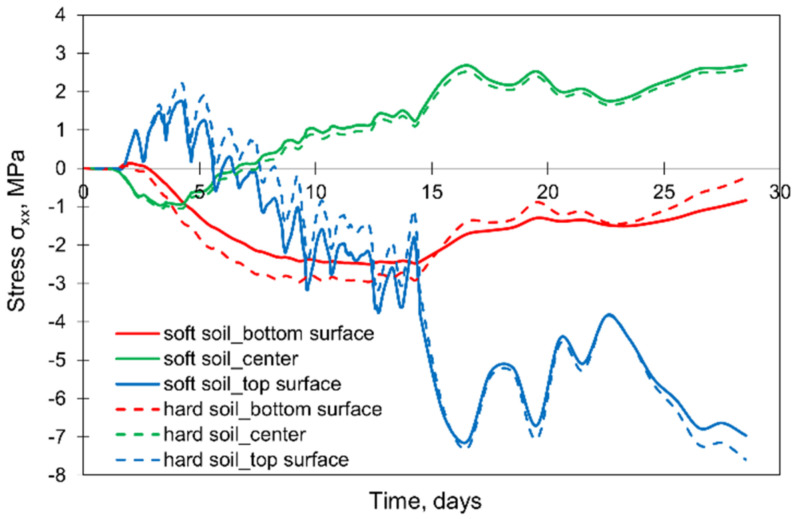
The effect of the soil stiffness on the development of stress $\sigma_{\mathrm{xx}}$.

However, the influence of soil stiffness turned out to be significant. It is noticeable in Figure 21 that the stresses at the top and bottom parts of the slab are higher in the model considering the soil of the higher stiffness. At the same time, the change of soil stiffness has a negligible impact on stresses in the centre of the slab. Hence, the stiffness of the subsoil significantly affects the stress values and special attention should be paid to its properties introduced to the FE model.

### 5.6. Importance of Shrinkage

**Figure 22:**
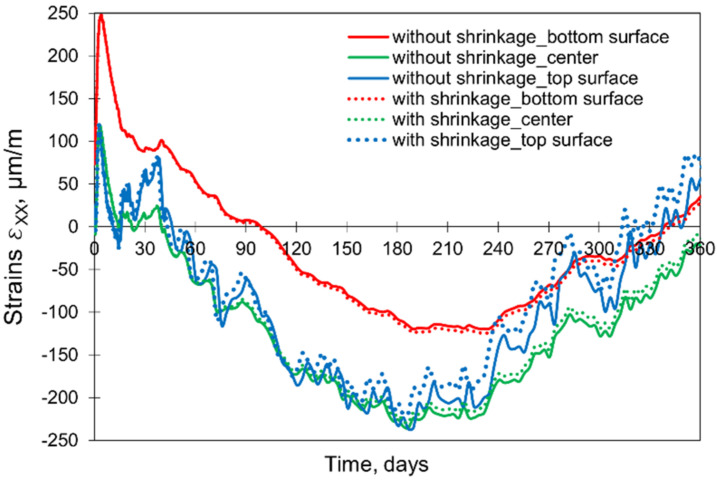
The influence of the shrinkage on the induced strains $\varepsilon_{\mathrm{xx}}$.

Considering the 28-day concrete curing, the stresses from the model involving both temperature and shrinkage are essentially identical to the stresses obtained from the model omitting shrinkage. It suggests that neglecting the shrinkage deformations at the early age period of concrete curing in the massive slab might be justified. A deeper analysis of this issue and the extension of the computation time to 365 days showed the differences between the corresponding developments of strains and stresses after 90 days. After that time, the effect of the shrinkage deformations on the induced total strains is noticeable at the top of the slab and successively progresses towards the centre of the slab (Figure 22). No influence of the shrinkage at the bottom of the slab is observed.

### 5.7. Effect of Material Model

In this section, the developments of the thermal stresses based on two material models, the viscoelastic material model without cracking consideration (used in Section 5.1, Section 5.2, Section 5.3, Section 5.4, Section 5.5, Section 5.6) and the material model with crack consideration, were compared.

Comparing the results of a 2 m thick slab obtained from the model without and with cracking, no significant discrepancies between the stress developments in the crucial points (top, centre, bottom) were noticed, and thus, the presentation of the graphs was omitted. Although the development of stresses in these three points did not suggest the cracking, the careful analysis of the results from the crack-based model indicated the occurrence of cracks in the cross-section located at 3.45 m from the symmetry plane of the slab. The cracks include the elements of the top and lateral surfaces of the slab with their widths reaching 0.01 mm. More details with the patterns of cracking are given in [32].

**Figure 23:**
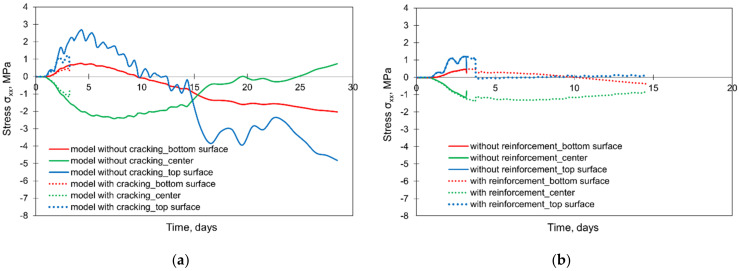
The effect of material model and reinforcement on the development of stress $\sigma_{\mathrm{xx}}$ in the slab with the thickness of 4 m: (**a**) comparison of material models, (**b**) effect of reinforcement in the crack-based material model.

The differences between the applied material models are clearly visible in the results of a 4 m slab (Figure 23a). First of all, the results from the crack-based model are available only until the 3rd day of analysis (corresponding to the 112th step). After that time, the calculations were terminated due to convergence failure. It can be noticed that the application of the material model considering cracking led to obtaining the lower values of stresses, especially at the top part of the slab. Furthermore, a dramatic drop in the stresses at the end of the analysis is observed, which indicates the occurrence of a macrocrack affecting the whole cross-section. Thus, the chosen material model has a crucial impact on predictions of concrete behaviour and possible cracking. The application of the crack-based material model gives more precise information about the potential cracking location and its range. Nevertheless, due to the more complex procedure of calculation, a quite common problem occurs to meet the convergence criterion. In this context, the application of the material without cracking model is more “user-friendly” as the time of analysis is shorter, and the convergence criterion is less daunting to acquire.

**Figure 24:**
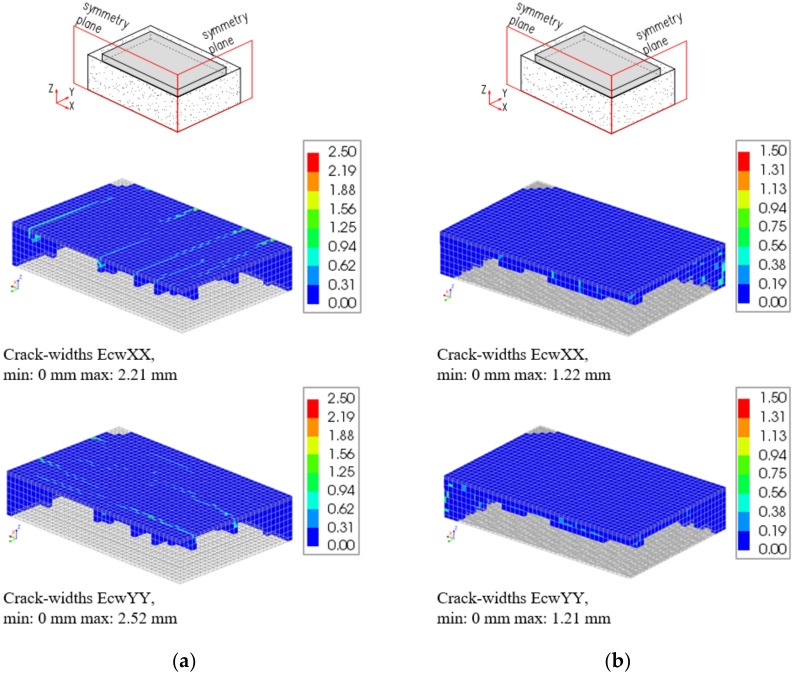
Patterns of local cracks distributions and widths in two directions (EcwXX, EcwYY) in the slab with the thickness of 4 m, corresponding to the 3rd day of analysis: (**a**) crack-based model without reinforcement, (**b**) crack-based model with reinforcement.

The second analysed issue was the need to consider the reinforcement in the FE model. Comparing the FE model without cracking, no differences in the obtained results were observed. In the crack-based model, the conducted comparative analysis indicates the important influence of reinforcement on the FE calculations (Figure 23b). First of all, the presence of reinforcement in the FE model improves the convergence conditions. The calculations were terminated later, after 14 days. Obviously, the reinforcement also improves the crack resistance of the slab, and this is evident in the results of the FE analysis. The cracking appears later (Figure 23b), and the widths of cracks are lower (Figure 24).

## 6. Conclusions and Outcomes for Engineering Practice

Due to the rapid growth in the availability and performance of computers, the use of FE analysis in civil engineering increases and will continue to increase in the foreseeable future. In this regard, the reliable model-based prediction of structural behaviour paves the way towards sufficient computational design and reduces the number of expensive laboratory experiments. The increased computing capabilities incline to model the structure and processes very precisely, albeit the direct consequence is the rapid growth of the number of input variables, the size of the model, and the time of FE analysis. Therefore, it is natural to provide all justified model simplifications which would not endanger the accuracy of the results. 

Keeping in mind a saying attributed to Albert Einstein that ‘everything must be made as simple as possible, but not one bit simpler’ the study is devoted to the optimal preparation of the FE model of massive foundation slabs at early ages. In the presented comparative studies, the most important aspects of the FE model were investigated by the corresponding modifications of the example of the real foundation slab. Both possible simplifications of the FE model (which do not affect the final results) and the crucial input data for the FE model (which are significant for the results) have been discussed. The results from the presented study, which may be useful for the engineering practice, as well as may be helpful as a reference for future FE simulations, are briefly summarized in Table 3.

## Figures and Tables

**Table 1:** Basic material data [29,30,32,33,34,42].

Material Property	Foundation Slab	Subsoil	Subbase	Reinforcement
E-modulus (28-day), GPa	34.4	30 × 10^−3^	27	210
Poisson’s ratio	0.2	0.2	0.2	-
Density, kg/m^3^	2349	2070	2400	-
Thermal expansion, 1/°C	1.2 × 10^−5^	1 × 10^−5^	1 × 10^−5^	1.2 × 10^−5^
Thermal conductivity, W/(m∙°C)	$3\;(\alpha_{H}$* = 0); $2.1\;(\alpha_{H}$* = 1)	1.4	1.7	-
Thermal capacity, J/(m^3^·°C)	2.3 × 10^6^	2.15 × 10^6^	1.95 × 10^6^	-
Yield stress, MPa	-	-	-	500

* $\alpha_{H}$—degree of heat development.

**Table 2:** Data for the layer projection of continuous casting in the validation of the FE model.

Number of the Layer	Thickness, m	Start Time, h	Steps
1	0.15	0	4 × 0.375 h
2	0.25	1.5	6 × 0.4167 h
3	0.35	4	8 × 0.4375 h
4	0.50	7.5	6 × 0.4167 h
5	0.35	10	4 × 0.375 h
6	0.25	11.5	4 × 0.25 h
7	0.15	12.5	12 × 0.5 h, 33 × 0.1 h, 14 × 0.24 h

**Table 3:** Summary of the results and recommendations for the FEA of massive foundation slabs at early ages.

Issue	Section	Results from FEA/Recommendations
Casting process	Section 4.2, Section 5.2	The precise reproduction of the casting process and its duration is recommended, especially in the thicker slabs, although it requires more laborious preparation of the FE model.
Computational domain	Section 4.3, Section 5.3	The recommended size of the finite elements is 15–20% of the slab thickness (central part); at the top and bottom surfaces, the height of the finite elements should be smaller due to the boundary conditions. The recommendation is valid for the models without cracking.The depth of the cooperating soil can be reduced to ~10 m.To set the horizontal size of the subsoil, preliminary calculations are suggested to determine the soil of the possible smallest size, which does not distort the results of the strains and stresses.
Ambient temperature	Section 4.4, Section 5.4	Forecast daily temperature changes (day/night) should be considered in the FE analysis because of the significant impact on the results. If the calculation needs to be simplified for any reason or the daily temperature changes are not available, the assumption of the maximum constant ambient temperature is preferable to estimate the maximum tensile stress at the top of the slab in the heating phase.
Properties of soil	Section 4.5, Section 5.5	The constant initial soil temperature can be assumed in FE analysis to skip the step of the analytical or numerical gaining of the input thermal data for the soil. The stiffness of the subsoil significantly affects the stress values, and this value should be properly introduced to the FE model.
Shrinkage	Section 4.6, Section 5.6	The shrinkage deformations at the early ages of concrete curing in the massive slab may be neglected. They may be relevant at later ages.
Material model	Section 4.7, Section 5.7	A crack-based model is recommended. It is more realistic and gives more precise information about the cracking risk, cracks locations, and ranges. The model without cracking gives only the knowledge on the values of the induced thermal stresses; nevertheless, it is more “user-friendly”, the time of analysis is shorter, and the convergence criterion is less daunting to acquire.
Reinforcement	Section 4.7, Section 5.7	Reinforcement may be neglected in the thermo-mechanical FE modelling without explicit simulation of cracking.In crack-based models, reinforcement should be considered. In this case, the reinforcement improves the convergence conditions and allows for a more realistic assessment of the time of the crack occurrence and its width.

## Data Availability

The data presented in this study are available on request from the corresponding author.
